# Effect of a Medication Adherence Mobile Phone App on Medically Underserved Patients with Chronic Illness: Preliminary Efficacy Study

**DOI:** 10.2196/50579

**Published:** 2023-12-11

**Authors:** Christa E Hartch, Mary S Dietrich, Deonni P Stolldorf

**Affiliations:** 1 School of Nursing Vanderbilt University Nashville, TN United States; 2 School of Nursing and Health Sciences Manhattanville College Purchase, NY United States; 3 Department of Biostatistics Vanderbilt University Medical Center Nashville, TN United States

**Keywords:** medication adherence, medication self-efficacy, mobile phone applications, medically underserved populations, mHealth, mobile health, app, apps, applications, underserved, adherence, survey, surveys, chronic illness

## Abstract

**Background:**

Medication adherence is vital in the treatment of patients with chronic illness who require long-term medication therapies to maintain optimal health. Medication adherence, a complex and widespread problem, has been difficult to solve. Additionally, lower-income, medically underserved communities have been found to have higher rates of inadequate adherence to oral medications. Even so, this population has been underrepresented in studies using mobile medication adherence app interventions. Federally qualified health centers provide care for medically underserved populations, defined as communities and populations where there is a demonstrable unmet need for health services. These centers have been reporting an increase in a more complex chronic disease population. Including medically underserved individuals in mobile health studies provides opportunities to support this disproportionately affected group, work toward reducing health disparities in access to health care, and understand barriers to mobile health uptake.

**Objective:**

The aim of this preliminary efficacy study was to evaluate the effects and feasibility of a commercially available medication adherence app, Medisafe, in a medically underserved adult population with various chronic illnesses seeking care in a federally qualified health center.

**Methods:**

Participants in this single-arm pre-post intervention preliminary efficacy study (N=10) completed a baseline survey, used the app for 2 weeks, and completed an end-of-study survey. The primary outcome measures were medication adherence and medication self-efficacy. Feedback on the use of the app was also gathered.

**Results:**

A statistically significant median increase of 8 points on the self-efficacy for adherence to medications scale was observed *(P=.*03, Cohen *d*=0.69). Though not significant, the adherence to refills and medications scale demonstrated a median change of 2.5 points in the direction of increased medication adherence *(P=.*21, Cohen *d*=0.41). Feedback about the app was positive.

**Conclusions:**

Use of the Medisafe app is a viable option to improve medication self-efficacy and medication adherence in medically underserved patients in an outpatient setting with a variety of chronic illnesses.

## Introduction

### Background

Inadequate adherence to medications in patients who require long-term medication treatment of chronic diseases is a global concern [1,2]. Inadequate medication adherence results in worsening symptoms, disease, complications, and death, resulting in a less healthy and productive society throughout the world [3]. This complex and widespread problem has been difficult to solve and requires innovative, multifaceted approaches to do so [4]. Providing effective patient education, involving patients in treatment decisions, considering health literacy, and providing audiovisual instructions as opposed to reading materials have been proposed to help patients adhere to their medication regimens [1]. Medication adherence also improves when drug regimens are simplified and medication costs, transportation needs, and available social supports are considered [1].

According to the Centers for Disease Control and Prevention, 4 in 10 adults in the United States have 2 or more chronic diseases, highlighting the need for interventions to improve medication adherence across disease spectrums and the lifetime disease progression trajectory [5,6]. Medication adherence is particularly important in medically underserved populations who receive care at federally qualified health centers (FQHCs). These patients have been found to experience higher rates of chronic conditions, and FQHCs are reporting growth in the rates of treating complex conditions [7]. Additionally, lower rates of medication adherence are reported in patients with lower socioeconomic status and patients who have multiple conditions [8].

One approach to enhancing medication adherence is the use of mobile smartphone technology, which offers new, different, and low-cost ways to interact with patients outside of the health care environment. Despite widely available, low-cost mobile smartphone medication adherence apps, a knowledge gap exists on the use of this technology and its impact on medication self-efficacy and medication adherence in medically underserved populations with a variety of chronic illnesses.

Medication adherence apps increasingly offer more interactive and customizable features, but the main function that can improve medication adherence and whether these interactive features contribute to user engagement over longer periods of time remain unknown [9]. Many of these apps offer features that encourage patient-centered behavioral approaches in an attempt to enhance user adherence to prescribed medication regimens [10]. A content analysis concluded that the use of established behavior change techniques (BCTs) in medication adherence apps is limited. The Medisafe app incorporates 5 of the 12 BCTs in its design, including action planning, prompts or cues, self-monitoring, feedback on behavior, and optional social support. Yet, the effects of BCTs on medication adherence remain unclear, particularly in medically underserved populations where there is potential to reduce health disparities [11-14]. Additionally, these behavioral approaches may vary depending on if the type of medication nonadherence is intentional or unintentional. Some features, such as app reminders, were found to be feasible for those who reported unintentional nonadherence, such as those who forget their medication [15]. In a study of chronic disease patients (N=24,017), 62% reported forgetting to take their medication [16]. In a study of noninsured, low-income patients with multiple chronic conditions, the most likely cause for nonadherence was forgetting (43.3%), and 48.7% reported nonadherence being unintentional [17]. These findings underscore the need to offer interventions to support these patients to adhere to their medications. Using a theory guided approach, discovering which behavioral concepts are impacted by the use of commercially available medication adherence mobile apps and understanding the uptake of these features by patients represent a gap in the literature [18]. In medication adherence research, the use of theory and conceptual frameworks has been underutilized, with one meta-analysis finding that less than 20% of the studies used theory to develop the intervention [19]. This seems to be even more true of interventions using medication adherence mobile phone apps [20]. A conceptual model that links the intervention inputs to the ultimate outcome, in this case medication adherence, is necessary to conduct valuable research [21].

### Purpose

A preliminary efficacy study was conducted to investigate if a publicly available high-quality medication reminder app (Medisafe) improved medication adherence and medication adherence self-efficacy in a population of medically underserved patients with chronic illness at an urban FQHC in the northeast United States. This study also evaluated the procedures for a future dissertation study and assessed recruitment and the feasibility and acceptability of the intervention. The aims of the study were as follows:

1. Our first aim was to investigate the preliminary effectiveness of using a medication adherence smartphone mobile app on patient medication adherence. We hypothesized that patients would report increased medication adherence with use of the mobile phone app.
2. Our second aim was to investigate the preliminary effectiveness of using a medication adherence smartphone mobile app on medication self-efficacy. We hypothesized that patients would report increased medication self-efficacy with use of the mobile phone app.
3. Our third aim was to examine the feasibility of using a medication adherence smartphone mobile app in a population of underserved patients with chronic illness. We hypothesized that patients would describe opportunities and challenges of using a smartphone mobile app and describe aspects of the app they found most helpful and those they found least helpful.

### Conceptual Model

**Figure 1:**
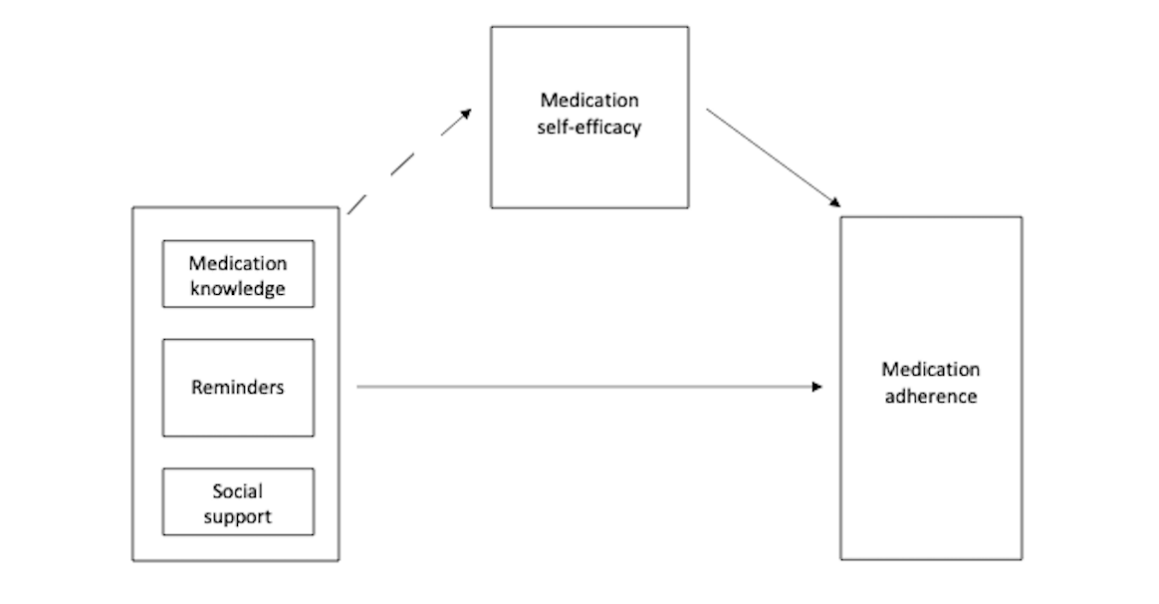
Conceptual model for the study.

The conceptual model of this study (Figure 1) is based on social cognitive theory (SCT), which is often used in medication adherence intervention research [19]. SCT posits that human action is caused by the interaction of behavior, cognitive and additional personal factors, and the external environment [22]. Self-efficacy, a focal belief of motivation and action, is posited to affect health habits directly [23]. Bandura [24] asserts that interactive technology, which mobile health falls under, may increase the impact of health promotion programs. Long-term adherence is facilitated by social support during early periods of personal change and maintenance [24]. Many of the constructs of the theory—for example, knowledge and sociostructural facilitators, such as reminders and social support—are integrated in some of the features of the Medisafe app. Applying SCT to this study, we hypothesized that medication knowledge, medication reminders, and medication social support affect medication adherence. We also looked at self-efficacy as a secondary outcome.

Medication knowledge is defined as patient-perceived knowledge about their medications [25]. Reminders are defined as automatically sent reminders without personal contact between the health care provider and patient [26]. Social support is broadly defined as the character and structure of a person’s relationships and involves support or assistance from family and friends [27]. We defined medication adherence as the extent to which a person’s behavior (eg, taking medication) corresponds with the agreed upon recommendations from a health care provider [28]. Lastly, medication self-efficacy is defined as the confidence the participant has in their ability to take their medications correctly in various situations [29].

## Methods

### Study Design

This study was a single-arm pre-post intervention preliminary efficacy study.

### Ethical Considerations

The Vanderbilt Institutional Review Board (no. 200530) approved the study, and the procedures followed were in accordance with the ethical standards of the responsible committee on human experimentation (institutional and national) and with the Helsinki Declaration of 1975, as revised in 2000.

### Setting and Recruitment

Participants were recruited from an FQHC in the northeast United States. The FQHC serves patients who are either insured by Medicaid or Medicare or are not insured. Some patients carry private insurance. Participants were recruited through health care provider referral for this study.

The inclusion criteria included: (1) adults aged 18 years and older, (2) patients who speak and understand English, (3) those who have access to an Android or iOS smartphone, and (4) those who take at least 1 medication for a chronic illness based on their computerized medical record at the health center. The exclusion criteria included: (1) those already using a medication reminder app or other electronic reminder system, such as phone alarms; (2) those owning smartphones that are not capable of downloading the app; (3) patients with severe dementia or serious mental illness; and (4) patients unable to use a mobile phone or the medication reminder software either physically or cognitively.

### Intervention

The medication adherence mobile app, Medisafe, is a publicly available, Health Insurance Portability and Accountability Act (HIPAA) compliant, no-cost medication adherence app. The app integrates the concepts of increasing medication knowledge, providing reminders, and offering an optional social support feature. HIPAA compliance is an important aspect of the app because some studies report smartphone users fear a compromise of their personal health information [18,30]. Additionally, this app can be used with patients with multiple chronic conditions, as opposed to only a specific chronic illness, which has been cited as an important design consideration and is an area for further study and development for medication adherence apps [18].

The Medisafe app includes adherence-tracking capabilities and the ability to communicate adherence reports with the health care provider. This smartphone app has been used in studies relating to hypertension [31] and coronary artery disease [32] and demonstrated improved medication adherence. The Medisafe app has not been studied in a medically underserved population with a variety of chronic illnesses in the United States.

### Data Collection

Data were collected using web-based surveys and secondary usage data from the Medisafe app. The participants completed a baseline survey and a follow-up survey after 2 weeks of app usage. REDCap electronic data capture tools were used to collect and manage the study data. REDCap is a secure, web-based software platform designed to support data capture for research studies [33]. The baseline survey consisted of the self-efficacy for appropriate medication use scale (SEAMS) and the adherence to refills and medications scale (ARMS) as well as the brief health literacy scale and patient demographic information. Based on patient preference, the baseline survey was administered either in person, via email, or in hard-copy form. The follow-up survey included the SEAMS and ARMS, questions asking for feedback regarding the Medisafe app, and 1 open-ended question for participant comments. If a hard copy was used, participant responses were double entered into REDCap to ensure accuracy. If the participant was unable to meet initially, contact information was collected and a date to come to the health center and complete the consent form, baseline questionnaire, and set up of the app was scheduled.

Secondary data collected by Medisafe were provided to the principal investigator (PI) in an Excel spreadsheet (Microsoft Corp) through Medisafe’s secure Google Drive platform.

### Study Measures

Several existing measures with established validity and reliability were used in this study. Brief descriptions of these measures and their respective times of assessment are shown in Table 1.

**Table 1:** Participant study measures.

Variable	Measurement	Time measured	Research aim	Items and subscales
Demographics	Gender, race, ethnicity, marital status, employment status, education, household income, type of health insurance, and current chronic illnesses	Baseline	N/A^a^	N/A
Self-efficacy for appropriate medication use	SEAMS^b^	Baseline, end of study	2	13
Medication adherence	ARMS^c^ adherence component	Baseline, end of study	1	12
Intervention process	N/A	End of study	3	Varies

^a^N/A: not applicable.

^b^SEAMS: self-efficacy for appropriate medication use scale.

^c^ARMS: adherence to refills and medications scale.

#### Self-Efficacy for Appropriate Medication Use

The SEAMS measures self-efficacy for medication adherence. It has been reported to be a valid and reliable global (continuous) 13-item scale, with a possible score of 13-39 (Cronbach α=.89) [29]. Participants are asked their level of confidence about taking medications correctly using response options of 1 (not confident) to 3 (very confident). Responses are summed to arrive at a total score, with higher values indicating higher levels of self-efficacy for medication adherence. The SEAMS has been reported to be valid and reliable in low-literacy populations with chronic disease [29]. Scores in our study had a reliability coefficient of α=.94.

#### Medication and Refill Adherence

Participants completed the ARMS to measure both medication and refill adherence. The ARMS is a valid and reliable global (continuous) 12-item scale. Responses are assessed on a 4-point scale ranging from 1 (none) to 4 (all of the time). Item responses were summed to generate a total score of 12-48. Lower ARMS scores indicate better adherence. The ARMS has been tested in a low-literacy population with chronic disease [34]. Prior internal consistency of the measure scores have been reported to be α=.81 [34]. The scores in this study had an α=.84.

#### Intervention process

Satisfaction and open-ended questions were used to ascertain opportunities and challenges of using the smartphone app and aspects of the app that participants found most and least helpful. For the feedback portion of the follow-up questionnaire, questions were asked about the usefulness and the ease of use of the app using a 5-point Likert scale (1=disagree strongly, 2=somewhat disagree, 3=neutral, 4=somewhat agree, and 5=strongly agree). Additionally, questions included how often certain features were used and how often the participants had technical issues. Feedback was also collected on whether or not specific additional features were used (yes or no), and if yes, participants were asked about their usefulness using the aforementioned Likert scale. The last question provided an opportunity for participants to provide any further comments or suggestions about the app in an open-ended format.

### Study Procedures

Clinic staff referred patients who met the eligibility criteria to the PI, who met with patients to discuss the study and obtain informed consent. After providing consent, the participants completed the baseline questionnaire either as a hard copy survey or web-based survey using an iPad (Apple), depending on their preference. The PI then assisted the participant with downloading and setting up the Medisafe app on their smartphone at the clinic. Once the app was installed, the medications were entered into the Medisafe app using the most recent medication list, which was obtained from the electronic medical record and reviewed with the participant. If any medication discrepancies existed, the clinic staff were asked to review the medication list to resolve the discrepancy prior to entering the medication into the Medisafe app. Educational materials developed by the PI were used to educate participants on the use of the app.

If participants were having difficulty entering their medications, the process was demonstrated by entering one of their prescribed medications. The PI showed the participants how to access their list of medications in the app, edit a medication that was already in their list (eg, change the medication dosage), and access information about their medications in the app. Participants were then shown how to indicate that the medication was taken, skipped, or rescheduled once they received a reminder alarm to take their medication. Participants were also introduced to additional app features, such as the weekly adherence report and how to invite a family member or friend to be a Medfriend in the app.

At the end of the meeting, the participant was given the PI’s contact information and was shown a Medisafe email address they could access if they experienced any technical problems. Participants were encouraged to set up a Medfriend to receive reminders of missed medications as well as refill and morning reminders. Participant follow-up via telephone or text message occurred within the first week after implementation to ensure that the app was working properly and to answer any questions. Participants were again contacted after 2 weeks either via phone, a web link, or in-person to complete the final questionnaire, depending on participant preference.

### Analysis

Statistical software (IBM SPSS Statistics) was used for the quantitative summarization of the preliminary effects of the use of the app on the change in medication adherence (ARMS scores) and self-efficacy (SEAMS scores) from baseline to 2 weeks. Frequency distributions summarized nominal or ordinal data and used the median (IQR) due to the small sample size. Wilcoxon signed ranks tests were used to test for changes in the ARMS and SEAMS scores from baseline to 2 weeks. An α =.05 was used for statistical significance. Results were transformed to Cohen *d* effect size statistics. Descriptive statistics were used to summarize the responses to the quantitative feedback questions. Only 2 participants responded to the final open-ended question. Direct quotes (rather than results from thematic analyses) were included in this report due to the paucity of response to that question.

## Results

### Sample Characteristics

Of the 10 participants, the median age of the participants was 49 (IQR 35.75-60; range 34-64) years and men and women were equally represented (n=5 each). The majority of participants were non-Hispanic (n=6, 60%), married (n=6, 60%), and half were employed for wages (n=5, 50%), and 50% (n=5) had either some high school education or a high school diploma. Reported household income was low, with 40% (n=4) of the participants reporting an income of less than US $25,000 annually. Most participants (n=8, 80%) were either employed for wages or self-employed. The median brief health literacy score was 11.5 (IQR 8.75-14.25; range 3-15), indicating relatively high subjective health literacy (Table 2) [35].

**Table 2:** Participant characteristics (N=10).

Variable			Participants, n (%)
**Gender**			
	Men		5 (50)
	Women		5 (50)
**Hispanic or Latino**			
	Yes		4 (40)
	No		6 (60)
**Race**			
	White		3 (30)
	Black or African American		2 (20)
	Asian		1 (10)
	Other^a^		4 (40)
**Current chronic illnesses**			
	Hypertension		5 (50)
	Diabetes mellitus 2		2 (20)
	Hyperlipidemia		1 (10)
	**Other illnesses**		7 (70)
		Chronic migraines	1 (10)
		COPD^b^	1 (10)
		Hyperprolactinemia	1 (10)
		Hypothyroid	1 (10)
		Insomnia, anxiety, or depression	1 (10)
		Lupus or myositis	1 (10)
		Migraines or neck or shoulder pain	1 (10)
**Number of illnesses indicated**			
	1		6 (60)
	2		3 (30)
	3		1 (10)
**Marital status**			
	Married		6 (60)
	Separated		1 (10)
	Divorced		1 (10)
	Single or never married		1 (10)
	A member of an unmarried couple		1 (10)
**Employment status**			
	Employed for wages		5 (50)
	Self-employed		3 (30)
	Unable to work (disabled)		1 (10)
	I would rather not answer		1 (10)
**Education**			
	Some high school		3 (30)
	High school graduate, diploma		2 (20)
	Some college credit, no degree		2 (20)
	Bachelor degree		2 (20)
	Master degree		1 (10)
**Household income (US $ per year)**			
	10,000 to <25,000		4 (40)
	25,000 to <50,000		1 (10)
	50,000 to <75,000		2 (20)
	I don’t know/not sure		3 (30)
**Type of health insurance**			
	Sliding scale		2 (20)
	Medicaid		3 (30)
	Private		1 (10)
	Other		1 (10)

^a^Columbian (n=1) and Hispanic (n=3).

^b^COPD: chronic obstructive pulmonary disease.

### Medication Self-Efficacy and Adherence

**Figure 2:**
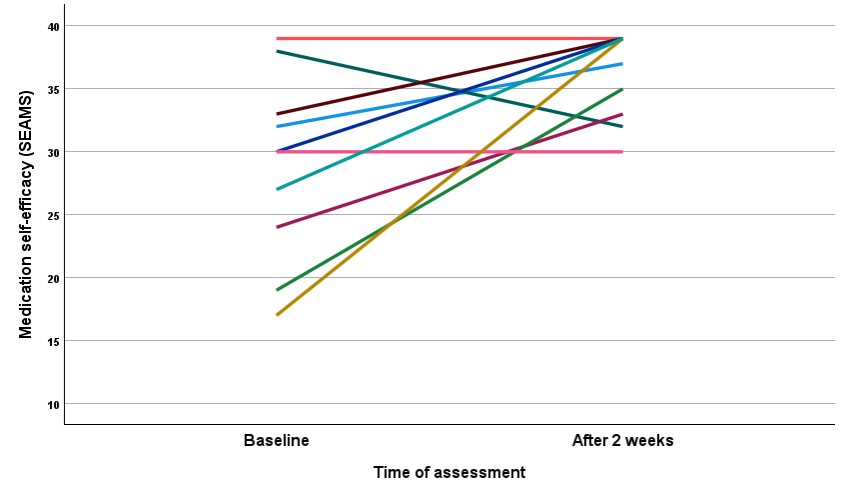
Medication self-efficacy pretest (baseline) and posttest (after 2 weeks) results. Different colors represent different patients. SEAMS: self-efficacy for appropriate medication use scale.

The median SEAMS score at baseline was 30 (IQR 22.75-34.25), with a range of 17 to 39. As shown in Figure 2, most of the participants reported an increase in self-efficacy after using the app for 2 weeks (median 38, IQR 32.75-39; range 30-39). This change was statistically significant (*P*=.03) and represented an effect size of 0.69 (Cohen *d*).

**Figure 3:**
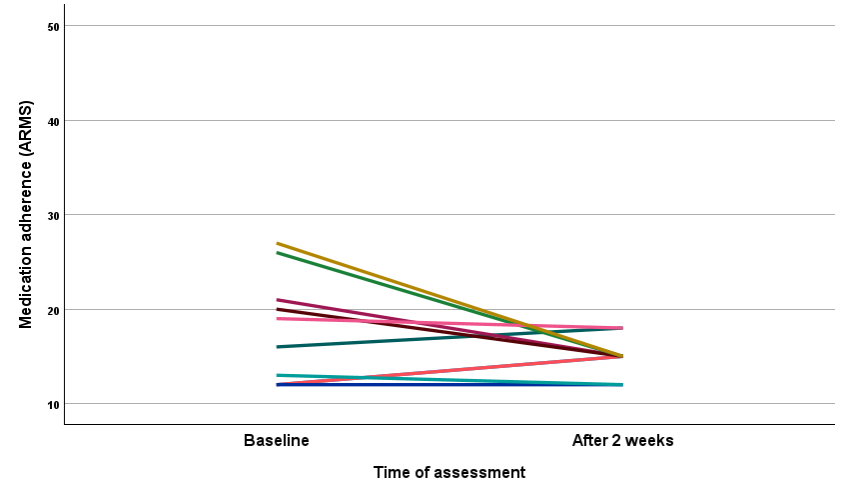
Medication adherence pretest (baseline) and posttest (after 2 weeks) results. Different colors represent different patients. ARMS: adherence to refills and medications scale.

A similar positive effect of app use in this study, although not significant, was observed for medication adherence (ARMS). Recalling that lower scores on the ARMS are indicative of better adherence, as shown in Figure 3, there was a general trend of increasing adherence, particularly for those participants with initial lower levels of adherence (ie, higher ARMS scores). The median score at baseline was 17.5 (IQR 12-22.25; range 12-27), and after 2 weeks of app use the median was 15 (IQR 15-16.5; range 12-18; *P*=.21; Cohen *d*=0.41).

### Participant Feedback

Feedback on the app was positive. Overall, 90% (n=9) of participants strongly agreed that they liked the app design, 90% (n=9) strongly agreed that it was useful to have a medication list on their smartphone, and 100% (n=10) found it useful to set medication reminders; however, only 40% (n=4) strongly agreed that tracking measurements was useful. None of the participants had difficulty with visualizing the app or problems with dexterity. There were 2 instances where participants needed to be corrected when entering medications. Additionally, 90% (n=9) of participants strongly agreed that the reminders helped them to remember to take their medications at the correct time each day and found it easy to use the app, while 80% (n=8) found it convenient to have the app. Only 1 participant received 3 or more reminders per day, while the others received 1 or 2 reminders per day. Although it had been set up in English, 2 of the participants opted to use the app in Spanish. Furthermore, 60% (n=6) of participants strongly agreed that having additional information about their medication in the app was useful, and 90% (n=9) would recommend the app to family or friends. Only 1 person reported technical issues and only 20% (n=2) reported using the refill reminder, with only 1 of them reporting that the refill reminder was helpful. Only 1 participant reported using a Medfriend and somewhat agreed that it was useful, and only 1 participant reported using the interaction checker and found it useful. One participant used the app to report an accurate list of medications to an emergency department physician and stated, “I would like to set my father up with it.”

At baseline, most participants preferred that the PI read the questions aloud and entered their responses into the tablet while they looked at a paper copy and either pointed to their response or gave their response verbally. However, some participants preferred to complete the follow-up survey using the email link.

## Discussion

### Principal Results

In summary, a statistically significant median increase of 8 points on the SEAMS was observed *(P=.*03; Cohen *d*=0.69). Adherence was generally high at baseline. Though not significant, the ARMS demonstrated a median change of 2.5 points in the direction of increased medication adherence *(P=.*21; Cohen *d*=0.41). Feedback about the app was positive.

### Medication Self-Efficacy

This study was the first to examine the influence of a widely available free medication adherence mobile app on medication self-efficacy in medically underserved patients with a variety of chronic illnesses. The findings showed that the intervention was efficacious in increasing participants’ medication self-efficacy from pretest to posttest. This finding aligns with the current literature indicating a positive association between medication self-efficacy and medication adherence [36-39]. Kjos et al [20] measured the concept of medication self-efficacy in relation to using a diabetes-specific medication adherence app. This single-group study showed no effect (Cohen *d*=0.04) on medication self-efficacy after 6 months in a group of privately insured outpatients with diabetes [20]. Notably, the study found a very small improvement in medication adherence (Cohen *d*=0.20). Upon further analysis, differential dropout rates suggested that younger patients with lower medication self-efficacy may have benefitted the most from this type of intervention and remained more engaged with the app [20]. The authors hypothesized that interventions that are more individualized and provide feedback and social support may have a greater effect on medication self-efficacy, although this remains unconfirmed [20].

### Medication Adherence

The current study showed an improvement in medication adherence, although the results were not statistically significant. Compared to the SEAMS scores, a slightly lower effect size was observed for the ARMS scores (Cohen *d*=0.41). The results of the effect of the app on medication adherence were similar to the average of other studies that researched medication adherence apps for specific conditions. There is quantitative evidence that there are generally small, positive correlations between using medication adherence apps with reminders and medication adherence in a variety of patients and settings. The Cohen *d* of the ARMS was similar to other studies measuring adherence using mobile phone reminders [30,32,40]. Additionally, this effect size was found to be larger than that in some previously presented studies [20,31,41]. Some studies found higher effect sizes [42-44]. These variations may be attributable to multiple factors, including measurements and various interventions. We found the largest increase in medication adherence in participants with lower adherence scores, which is similar to other studies that attributed smaller effect sizes to a ceiling effect related to having samples of more adherent patients [30,31,40].

Studies that have previously been conducted in low-income, vulnerable populations include that by Moorhead et al [40], which calculated the effect size (Cohen *d*=0.49) for patients taking the medication after “seeing” or “not seeing” the reminder. Chandler et al [42] found a larger effect size (Cohen *d*=1.96) in their multifaceted app intervention, which also included nursing outreach to Hispanic patients with hypertension. Additionally, 2 studies that looked at the effect of Medisafe on medication adherence in hypertension reported effect sizes of *d=*0.33 [31] and *d*=0.45 [32]; however, these studies were not conducted in medically underserved populations. The current study indicates that the Medisafe app can likely be used in medically underserved populations to improve medication adherence and medication self-efficacy.

Studies that assessed patient satisfaction and usability of the Medisafe mobile app intervention received high scores, with 75% of participants indicating they would continue to use the app [32]. The participants in this study similarly reported positive satisfaction and usability scores. Specifically, 90% of participants indicated that they will continue to use the app. The results indicated that reminders to take their medications were the most helpful and most used aspect of the app. Only 2 participants used the refill reminder and only 1 of them found it helpful. This may have been due to the duration of the study (ie, only 2 weeks) because in general, the patients had at least 1 month of medication supply before they needed to refill, which made it difficult to study this feature in retrospect. Additionally, this feature would be less helpful for participants who have intentional low adherence due to medication side effects or participant health beliefs. While some participants reported using the Medfriend option, the Medisafe data did not confirm this. It is possible that the person who was invited to be a Medfriend did not accept the invitation. There is a possibility that those who participated were more technologically inclined, since owning and using a smartphone was part of the inclusion criteria, which may have impacted favorably upon the results. However, 76% of low-income Americans, defined as those who earn less than US $30,000 annually, own a smartphone, and 27% of this population relies on smartphones for their internet service, up from 12% in 2013 [45]. Subjective health literacy scores were relatively high, and the results may have been different in those with lower subjective health literacy scores. No participants were excluded due to limited technology literacy or inadequate access to the internet, which supports the use of mobile technologies for patients who are interested in using them and the promise they may have in reducing health disparities.

### Limitations

Some study limitations should be noted. While the effect sizes for the SEAMS and the ARMS could have simply been due to regression toward the mean in this 1-sample study, the patterns observed for the majority of participants and the feedback received provide sufficient evidence to conduct a larger-scale randomized controlled trial. Such a trial will enable further understanding and a much more rigorous test of the efficacy of a widely available medication adherence mobile app in populations of medically underserved adults with chronic illnesses. This study may have a risk of bias as some of the participants may have been known to the clinic staff who introduced the study. This was not measured, however, and it is unknown how many of the participants fell into this category. There was no loss to follow-up in this study, which is a strength of this research.

### Conclusions

This study demonstrated that an existing, widely available, and free medication adherence mobile app can be used to improve medication adherence and medication self-efficacy in medically underserved populations with chronic illnesses. Patients rated the app positively, were satisfied with the app, and planned to continue using it in the future. The study findings suggest that the use of a mobile app might be feasible to address and improve medication self-efficacy and medication adherence in medically underserved populations.

## Abbreviations

ARMS: adherence to refills and medications scale
BCT: behavior change technique
FQHC: federally qualified health center
HIPAA: Health Insurance Portability and Accountability Act
PI: principal investigator
SCT: social cognitive theory
SEAMS: self-efficacy for appropriate medication use scale
